# Applying modified coot optimization algorithm with artificial neural network meta-model for building energy performance optimization: A case study

**DOI:** 10.1016/j.heliyon.2023.e16593

**Published:** 2023-05-25

**Authors:** Xiaoming You, Gongxing Yan, Myo Thwin

**Affiliations:** a College of Civil Engineering, Chongqing Vocational Institute of Engineering, Chongqing, 402260, China; b School of Intelligent Construction, Luzhou Vocational and Technical College, Luzhou, 646000, Sichuan, China; c Luzhou Key Laboratory of Intelligent Construction and Low-Carbon Technology, Luzhou, 646000, China; d Yangon Technological University, Myanmar; e College of Technical Engineering, The Islamic University, Najaf, Iraq

**Keywords:** Artificial neural network, Building performance optimization, EnergyPlus™, Meta-model, Modified coot optimization algorithm, Multi-criteria optimization, Residential building

## Abstract

Today, an important problem of the building energy performance area is carrying out multi-criteria optimizations of real building designs. To solve this problem, a new method based on a meta-model is proposed in this study. Hence, the EnergyPlus™ is used as the simulation tool for the performance simulation of the building, then a couple of the multi-criteria Modified Coot Optimization Algorithm (MCOA) dynamically combined with the artificial neural network meta-models (ANN-MM) are employed. For the sample generation applied for training and validation of ANN meta-models, an optimum way is presented by this method to minimize the whole building energy simulations needed for their training, and validate precise results of optimization. Moreover, the method is used for the thermal comfort and energy efficiency optimization of a real house to achieve the optimum balance between the heating and cooling behavior of the case building. 12 effective design variables of this case study are selected. Also, the achieved results are put in comparison with the “true” Pareto front found through an optimization method based on simulation performed for more validation. It is assumed that 1280 points are adequate in this case study to obtain precise results on the Pareto set. Thus, 75% of the required simulations’ number based on physics has been saved by this size of sample considering the 5120 applied in the method based on simulation. Consequently, the optimum Pareto set of a real multi-criteria building efficiency optimization problem is achieved by the proposed method and accurate results are achieved.

## 1 Introduction

**Fig. 1:**
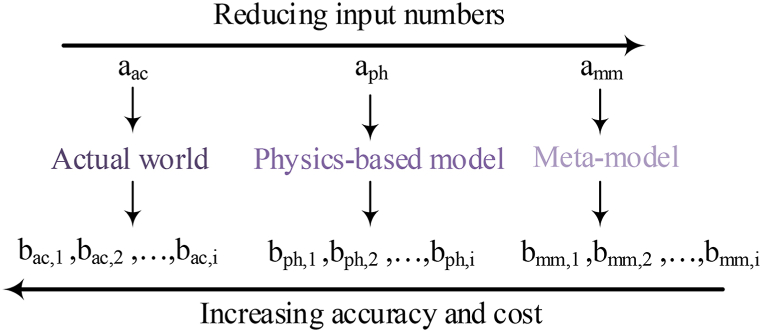
The association of computational cost and accuracy for various building design methods.

Decrease GHG emissions, the residential sector has a significant role because it considerably affects global warming [1]. To enhance the low-carbon way of life household energy use models and GHG emissions have been investigated by research workers in various countries [2]. The energy-effective design of the buildings has a considerable effect on the decreases in energy consumption [3]. To achieve this target, the building behavior (energy use, and thermal and visual comfort) prediction is needed, and the physics of the building should be investigated from an integral perspective including the performance of the heat, installed equipment, and mass transfer process, and the association of them with the behavior of the users in the building [4]. Current buildings in Morocco are frequently designed without considering the climatic conditions. The best temperatures for proper efficiency between the room's thermal comfort and the building energy consumption for air conditioning in summer and for heating in winter are 26 °C and 20 °C, respectively. A common problem of Moroccan traditional houses involves moisture. Moreover, intolerable variation in temperature between winter and summer is a problem. Mainly, the factors including the climate, the frontages orientation and the sun shining absence in the patio in winter, the air renewal and the ventilation, and the building materials' thermal quality are the reasons for these thermal discomfort problems [5]. To help the designers, Building Performance Simulation (BPS) can be an efficient tool [6]. By this method, it is possible to study different designs to perform particular targets like decreasing environment-related effects and energy use, or enhancing interior thermal comfort [7]. This uncertainty inherent is the main challenge in building retrofitting and simulation, specifically as financial and investment risks are more and more involved in building performance simulation achievements [8]. The evaluation of multi-variable problems by automated computer-based technologies has enabled investigators to identify optimum multiple-criteria solutions in vector space with n dimensions [9]. Then, genetic algorithms (GA) extensively have been employed, which enable obtaining further optimized and effective solutions [10]. However, genetic algorithms have the main constraint [11]. The optimization comes to be difficult when the fitness function assessment is a time-consuming process for buildings with medium/large sizes, particularly if it relates to a real case study with conflicting multiple criteria [12]. A meta-model (MM) or surrogate model (SM) is a mathematical algorithm indicating inputted and outputted associations displayed by other further complicated models (models based on physics) [8]. As a major benefit, these techniques can be established by a small data set achieved through a design simulation based on physics at properly chosen design items [13]. Moreover, their formulations can be defined analytically, and their application is easy [14]. Thus, after the meta-model has been trained with the appropriate input and output sets obtained as a model result based on physics, it can be applied for the estimation of the outputs (environmental performance, energy use, weather condition, comfort index, and so on) achieving from other inputted values not contained in the sample of training [15]. Fig. 1 shows the association of computational cost and accuracy for various building design methods [16].

To solve multi-objective optimization by meta-models some research has been carried out [17]. For example, in Ref. [18], to obtain the lowest heating and cooling loads a multi-objective optimization has been implemented in a residential case combined with “phase-change materials” (PCMs) [19]. “Grouped Method of Data Handling” (GMDH) kind of ANN, numerical modeling by EnergyPlus, and NSGA-II were used to achieve the objective of the paper. In Ref. [20], the influence of elliptical shape sections of confined concrete reinforced with Carbon Fiber Reinforced Polymer (CFRP) and steel tube on axial load-carrying capacity has been investigated by ANN modeling and Finite Element (FE) in Abaqus. In Ref. [21], an approach for the optimization of the equal thermo-physical properties of the outer walls of a house to enhance its thermal effectiveness is proposed. An artificial neural network and the genetic algorithm NSGA-II are coupled to decrease the computational conditions. A comparison of the optimum solutions with the ones from single-objective optimization indicated the significance of implementing multiple-objective optimization by application of an aggregative approach and a constraint problem in GenOpt. In another work [22], “Building Performance Optimization” (BPO) is applied to a recently constructed complicated construction design. An SM method improved by an ANN has been applied to decrease the time of computation. Four multiple-objective algorithms’ performance has been assessed and the results showed that NSGA-II has the optimum performance in the case study.

### 1.1 Research significance

ANNs are generally applied as the meta-model in the optimization methods based on the meta-model [23]. This has been trained by a small representative sample achieved by any sampling technique [24]. Nevertheless, it is not possible to define the correct training sample size previously due to that it depends on the problem [25]. Indeed, until now, trial and error is used to define this size for a particular case study building. This may undermine the benefits of this approach. Also, this specific problem has not been precisely studied with multiple-criteria building performance optimization considerations until today [26]. In this respect, proposing an efficient approach to achieve a solution for multi-objective building performance optimization problems by a new method based on a meta-model is the major target in this paper for the minimization of the overall number of physics-based building performance simulations needed. To develop the samples applied to train and confirm the meta-models based on Artificial Neural Network, the Augmented Latin Hypercube (ALH) sampling method is suggested. This led to the minimization of the building energy simulations number needed for training them and validating precise multiple-criteria optimization achievements. To verify the present method, it is used to a complicated multiple-criteria optimization problem (MCOP) of a real building with higher than 10^8^ feasible models. To achieve the results of the building performance simulation for each sample for training the meta-models based on Artificial Neural Network, the EnergyPlus™ simulation tool was employed. Then, they are dynamically combined with the multiple-criteria Modified Coot Optimization Algorithm (MCOA) to obtain the optimum balance between heating and cooling behavior of the case building. Moreover, the achieved results are put in a comparison with the “true” Pareto front achieved by an optimization method based on simulation is performed for validation.

## 2 Materials and methods

### 2.1 Multiple-criteria BPO

The BPO is mathematically presented as an MCOP [27]:

(1) $$\min\;f_{a}\left(y\right)\;a=1,\ldots ,n$$

where, $f_{a}$ stands for a particular objective and y defines *k* building design variables set. The trade-off among the objectives $f_{a}\left(y\right)$, $f_{b}\left(y\right)$, …, $f_{n}\left(y\right)$ is achieved by the solution of the multiple-criteria problem (1). When there is a mutual conflict among these objectives, a multi-dimensional space is formed beside the typical decision space. Thus, the solution to this problem is a group of solutions that are not dominant. This solution set establishes the Pareto set with only two objectives. As aforementioned, BPO is tackled through an optimization method based on simulation, which couples a building performance simulation tool with a computational optimizer. As regards bio-inspired optimizers, most of them have been presented to find a solution for truly multiple-criteria problems. In this study a multi-objective version of MCOA called MO- MCOA has been used to achieve the optimum balance between the heating and cooling behavior of the building. The main reason for using MCOA is that it has high efficiency in both accuracy and precision in solving optimization problems.

### 2.2 Meta-model-based optimization method

**Fig. 2:**
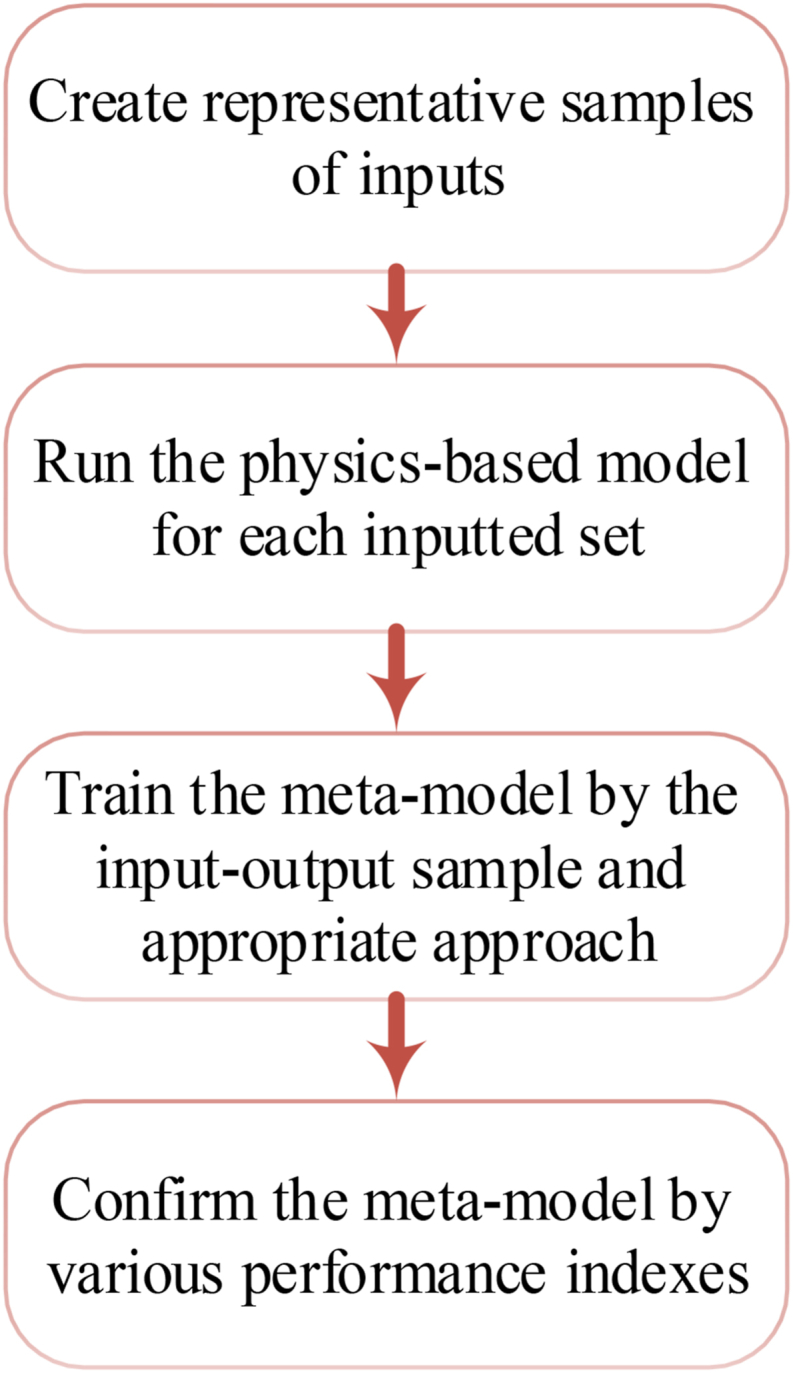
The workflow of the generation of meta-models.

In building performance simulation uses due to the high performance of meta-modeling over tasks with numerous simulations such as optimization, uncertainty, and sensitivity evaluations of the real case studied, Meta-modeling has been noticed widely by designers. The workflow of the generation of meta-models is defined in Fig. 2.

**Fig. 3:**
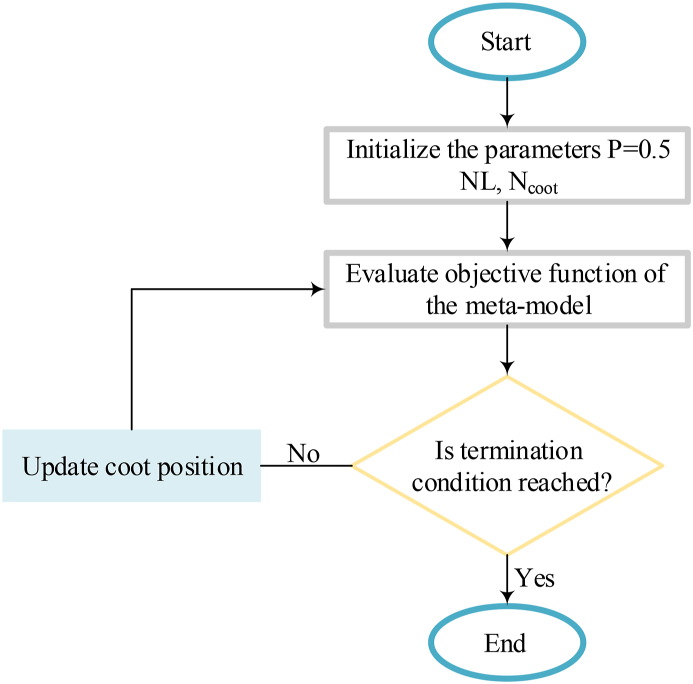
The algorithm flowchart of the optimization method based on a meta-model.

The meta-model is applied as an SM method for the quick building performance simulation after these phases are performed. Fig. 3 depicts the algorithm flowchart of the optimization method by the meta-model.

In the techniques based on meta-models, the performances in optimization algorithms need almost the same time as before with an additional cost for training the meta-model and a small cost for assessments of it. Moreover, a limited number of building performance simulations based on physics in a particular building is appropriate to decrease the cost of computation of the total optimization process. Notably, as the complicatedness of the problem gets more, the cost of computation for training and assessment of the meta-model does not considerably change. However, the costs of simulations based on physics necessary for the generation of the training sample get more as well as the problem's complicatedness to a limit point.

From this perspective, two issues are raised; the first one is that is it possible to decrease the number of building performance simulations based on physics in the method based on meta-model for real buildings optimization and the second one is the size of the training sample to verify the needed accuracy for the optimization process. For multiple-criteria problems, it is not easy to respond to these issues because the accuracy should be obtained over the total Pareto set. Hence, it is aimed to discuss these issues in this paper to present an effective and workable method based on a meta-model to implement multiple-criteria BPOs.

### 2.3 Case study building description

**Fig. 4:**
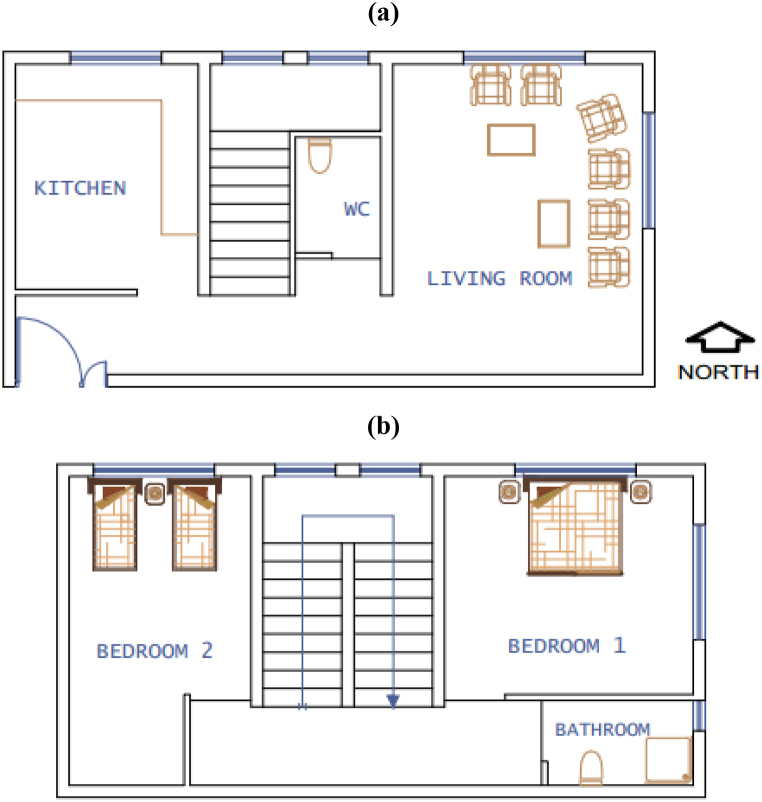
The plan of the single-family house. (a: ground floor, b: first floor).

A typical single-family house is selected to employ the methodology. The house has two stories and the area of the house is equal to 85 $m^{2}$. As shown in Fig. 4(a and b), this building is a detached house.

The considered building is located in Porto Alegre, Brazil, with latitude and longitude equal to 30.0368° S, and 51.2090° W, respectively. Table 1 states the major features of the house ($C_{0}$).

**Table 1:** The major features of the typical case study building.

Element	Features
1st floor	Concrete and ceramic floor
Exterior and interior wall	Hollow wall brick with mortar finish
Exterior solar absorptivity (exterior walls)	0.7
Roof	Ceramic tile, air gap, and concrete liner
Azimuth	0 (north-faced)
Windows	Simple clear glass with a 3 mm thickness
Fraction of shading (windows)	25%
Window area fraction for natural ventilation	30%
Rate of infiltration (doors and windows)	0.02 kg m/s

**Fig. 5:**
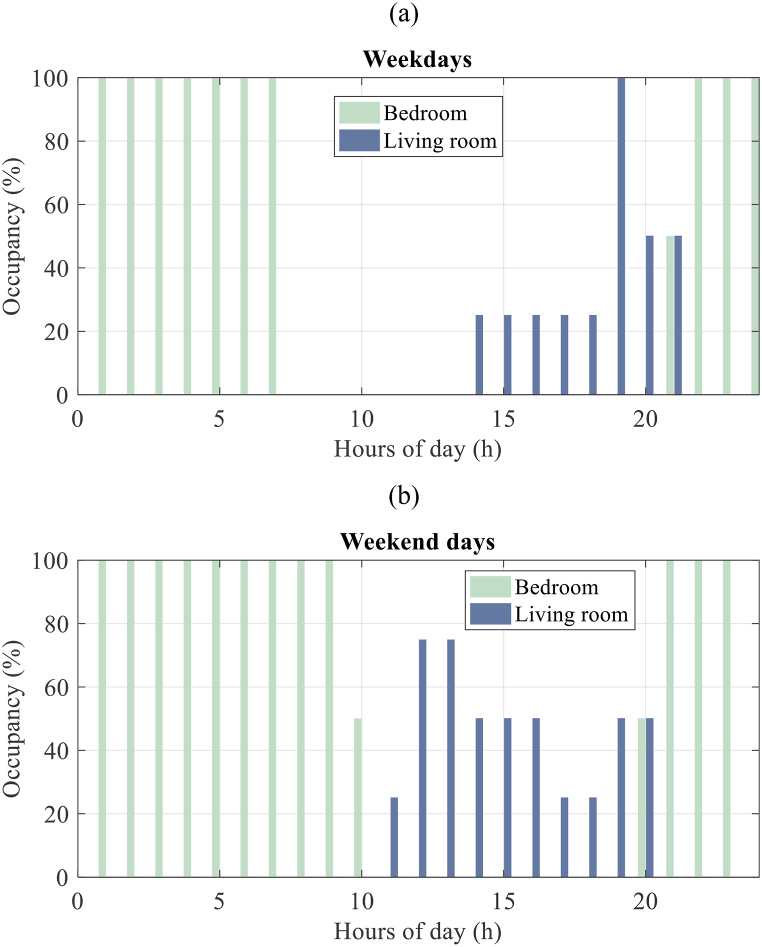
Occupancy schedules of weekdays (a) and weekend days (b) for the bedrooms and the living room.

For the assessment of energy and thermal performance and alternative designs of the house, EnergyPlus™ [28] is employed. The house has 8 thermal zones including a living room, 2 bedrooms, a kitchen, 2 bathrooms, a staircase, and corridors as shown in Fig. 4. The house is designed for four people. The occupancy schedules of the living room and bedroom are represented in Fig. 5 (a, b). In line with ASHRAE [29] standards, all rooms have their related indoor heat load as regards the occupants, the equipment, and the lighting.

For each room, natural ventilation by doors and windows is considered when the interior temperature ($T_{in}$) is more than the exterior temperature ($T_{ex}$), where $T_{ex}$ is higher than 20 °C. Moreover, an air conditioner is considered for the bedrooms, which heats the room if $T_{in}$ is equal to or lower than 18 °C and cools the room when $T_{in}$ is higher than 26 °C at the occupied time.

#### 2.3.1 The performance measurements of the building

The building performance is assessed in the bedrooms and living room. A “discomfort index” by the heating and cooling “degree-hour” is used for the performance measurement of the living room that is considered to be only naturally conditioned. This can be calculated as follows [30]:

(2) $$\left\{ \begin{array}{c} D_{h}=\sum_{t}(T_{l}\left(t\right)-T_{o}\left(t\right)\;D_{h}\geq 0\\ 0\;D_{h}<0 \end{array}\right.$$

(3) $$\left\{ \begin{array}{c} D_{c}=\sum_{t}(T_{o}\left(t\right)-T_{u}\left(t\right)\;D_{c}\geq 0\\ 0\;D_{c}<0 \end{array}\right.$$

where, $T_{l}$ is the lower acceptable temperature. $T_{o}$ denotes the operative temperature in the room at time t. $T_{u}$ defines the upper acceptable temperature. Except for the time the room is unoccupied, the previous sums range is a total year. $T_{l}$ and $T_{u}$ can be considered as the 80%-admissibility range as follows [31]:

(4) $$T_{u}=0.31T_{pma\;\left(o\right)}+21.3{}^{\circ}C$$

(5) $$T_{l}=0.31T_{pma\;\left(o\right)}+14.3{}^{\circ}C$$

where, $T_{pma\;\left(o\right)}$ stands for the “prevailing mean outdoor temperature”. This is considered as the local dry-bulb temperature's monthly mean.

The performance in bedrooms is calculated through the yearly energy use of the air conditioners for cooling ($E_{c}$) and heating ($E_{h}$) considering that in this room thermal comfort is artificially applied when it is needed. Then, the air conditioner is on when natural air conditioning is not acceptable. The hybrid ventilation availability manager in EnergyPlus™ is used for the integrated use of active and passive cooling/heating approaches.

In the bedrooms, Packaged Terminal Heat Pumps (PTHPs) model is considered for the air-conditioners. When these rooms are occupied at a specified time t, the air-conditioners work to heat the room when the room temperature ≤18°C and to cool when the room temperature ≥26°C.

#### 2.3.2 Objective functions

To enhance the performance of the building at cooling and heating times and by the aforementioned indices of thermal and energy performance, the objectives are defined as follows:

(6) $$f_{c}\left(z\right)=w_{d}\frac{D_{c}\left(z\right)}{D_{c}\left(z_{0}\right)}+w_{e}\frac{E_{c}\left(z\right)}{E_{c}\left(z_{0}\right)}$$

(7) $$f_{h}\left(z\right)=w_{d}\frac{D_{h}\left(z\right)}{D_{h}\left(z_{0}\right)}+w_{e}\frac{E_{h}\left(z\right)}{E_{h}\left(z_{0}\right)}$$

where, $w_{d}$ and $w_{e}$ stand for the weighting factors where $w_{d}+w_{e}=1$. $z_{0}$ defines the design variables set for $C_{0}$. $w_{d}=w_{e}=0.5$, assuming that the occupation time of the bedrooms (included in $E_{c}$ and $E_{h}$) and the living room (included in $D_{c}$ and $D_{h}$) have the same extension. It is noteworthy that $f_{c}$ and $f_{h}$ are set as the weighted sum of sub-objectives $D_{c}$, $D_{h}$, $E_{c}$ and $E_{h}$. In conclusion, the present multiple-criteria optimization problem can be defined as follow:

(8) $$\min\left[w_{d}\frac{D_{c}\left(z\right)}{D_{c}\left(z_{0}\right)}+w_{e}\frac{E_{c}\left(z\right)}{E_{c}\left(z_{0}\right)}\;w_{d}\frac{D_{h}\left(z\right)}{D_{h}\left(z_{0}\right)}+w_{e}\frac{E_{h}\left(z\right)}{E_{h}\left(z_{0}\right)}\right]$$

#### 2.3.3 Description of the building design variables

For the case study house optimization, 12 design variables are selected that are the most effective ones based on the sub-objectives. Morris's approach to sensitivity evaluation was used to analyze the significance of inputted parameters [32]. Table 2 states the selected continuous variables and their discretization of them. Note that after discretization, the width of the window changes to a categorical parameter.

**Table 2:** The description of the selected continuous variables.

Variable	$V_{1}$	$V_{2}$	$V_{3}$	$V_{4}$	$V_{5}$	$V_{6}$	$V_{7}$
Definition	Azimuth	Shading size of the window	Solar absorptance (exterior walls)	Infiltration rate of windows	Infiltration rate of doors	Fraction of window area for natural ventilation	Window width
Max	315°	100%	0.9	2 × 10^−2^ kg/m/s	2 × 10^−2^ kg/m/s	50%	4
Min	0°	25%	0.3	10^−5^ kg/m/s	10^−5^ kg/m/s	10%	1
No. levels	8	4	4	4	4	5	4
Step	45°	25%	0.2	6*.*67 × 10^−3^ kg/m/s	6*.*67 × 10^−3^ kg/m/s	10%	1

Table 3 illustrates the other categorical variables such as types of windows, roofs, walls, etc.

**Table 3:** Categorical design variables.

$V_{8}$	
Definition	Level
Exterior walls	- Wood with an air gap
	- Double hollow brickwork layers with insulation and mortar finishing
	- Concrete
	- Double concrete block, insulation, and cement-plaster finishing
	- Hollow brickwork layer, mortar finishing
	- Wood, insulation, and plaster finishing
	- Concrete block, cement-plaster finishing
$V_{9}$	
Definition	Level
Roof	- Concrete and hollow ceramic block, plaster ceiling
	- Ceramic tile, air gap, and wood or concrete liner
	- Concrete, plaster ceiling
	- Ceramic tile, insulation, air gap, and concrete or wood liner
$V_{10}$	
Definition	Level
Window	- Single clear 3 mm or 6 mm glass
	- Double clear 3 mm or 6 mm glass, an air gap
$V_{11}$	
Definition	Level
Interior walls	- Wood with insulation and plaster finishing
	- Hollow brickwork layer, mortar finishing
	- Concrete
	- Wood, an air gap
	- Concrete block, cement-plaster finishing
$V_{12}$	
Definition	Level
Floor-type (1st)	- Concrete, wood, or ceramic floor
	- Insulation, concrete, ceramic floor

In this study, the continuous variables have been discretized while the categorical ones are inherently discrete, in other words, we are allowed to use only “levels” or definite discrete values of variables, mostly to clarify the limitations of the case study-building procedure.

### 2.4 Meta-model-based method

**Fig. 6:**
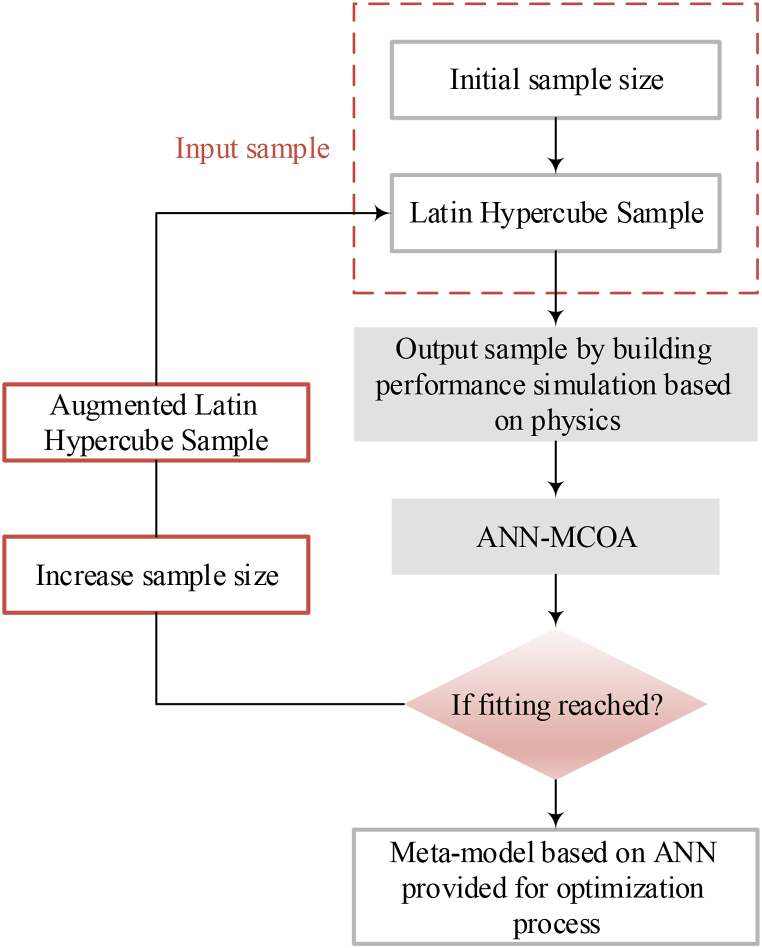
The workflow of the method suggested developing the meta-model based on the ANN.

An optimization model based on a meta-model by MCOA as shown in Fig. 3 is presented here to achieve a solution defined in Eq. (8). In recent years, meta-models based on ANN have been used for building performance simulation due to their great performance. Therefore, an ANN-based meta-model is employed for the house-building performance evaluation in the optimization process. The workflow of the method suggested to develop the meta-model based on the ANN is depicted in Fig. 6.

Latin hypercube (LH) sampling [33], which is a statistical approach to generate a “near-random sample” of variable amounts from a multiple-dimensional distribution has been used to achieve a representative sample of the inputs for training and validation (T&V). To compute the outputs set, a simulation based on physics by EnergyPlus™ is used. Then, the T&V of the ANN-MM is performed. The meta-model can be applied in the optimization process if the minimum errors of the meta-model based on ANN in the validation sample are reached, else, the sample size should be increased and the equal process should be performed again until the minimum errors are reached.

### 2.5 Meta-models based on ANN

The steps that define the general training process of ANN-based meta-models are including ANN stimulation by an environment, alterations in the free parameters of ANN resulting from the simulation, and an answer to the environment in a new way because of the alterations that appeared in the artificial neural network internal configuration.

*Sampling approach;* a representative approach such as Latin Hypercube (LH) sampling [33] can be used to create input samples for sampling approaches. Then, the sample is split into validation and training samples. The main sample representativeness is no more assured in the achieved new samples, which is the major drawback of this method. A*“k-fold”* cross-validation approach is employed in the process of training to modify this. The steps of this method is including a) the training sample division into *k* folds, all with equal size, b) choosing a fold as the sample of validation and with the rest data, the meta-model is trained, c) Repeating the former phase *k* times, by a various subdivision for validation of each time, and d) the model performance is measured by averaged performance over each sample of validation in *k* times of repetitions. Nevertheless, to prevent a high increase in the computing cost, the parameter *k* must be selected precisely, however, still, the optimum achieved samples of validation and training are not representative as same as the main one created by the LH sampling approach.

Only to train and create an additional sample for meta-model validation, an alternative method [34] uses the sample. As represented in Fig. 6, both samples of the training and the validation are built independently based on this concept. Therefore, for training and validation of the meta-model, two representative samples have been achieved, which decreases the cross-validation times and permits the meta-model precision to be confirmed. It should be noted that with the LH sampling approach, seven representative samples are created with an overall 5120 simulations due to the sample size enlargement is possible and use again the simulation achievements of the former sample [35]. In addition, all the samples were run to confirm the achievements systematically by comparing the real Pareto set achieved by the method based on simulation in addition to considering the appropriate stopping criteria. About the samples of validation, the equal process is applied, however using a 10% training size, increasing the other seven samples of LH sampling of 8, 16, 32, 64, 128, 256, and 512.

**Fig. 7:**
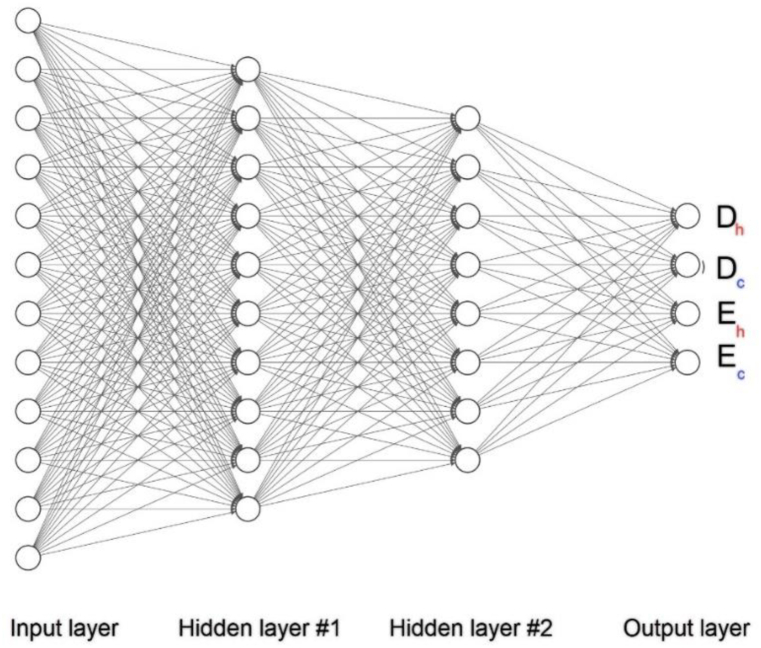
The ANN architecture.

***ANN architectures;*** a feed-forward multiple-layer artificial neural network is used herein. The architecture of the ANN has been depicted in Fig. 7.

Artificial neurons exist in each output and hidden layer interconnecting through adaptive weights, which have been calibrated via a training procedure with input-output data. To provide reliable results min-max normalization in the range between 0 and 1 has been utilized. 12 design variables according to the inputs of ANN are considered, which has been reported in Table 2, Table 3 For the computation of the two contradicting objectives, regarding the outputs of ANN, four sub-objective $D_{c}$, $D_{h}$, $E_{c}$, and $E_{h}$ are assumed herein.

***Training and validation;*** the outputs set is achieved by the achievements of the simulation tool considering a sample size for the inputs set defined by the Latin hypercube sampling technique. Thus, the process of training is performed by these input/output data. In this procedure, “overfitting” is a usual problem. This happens if the artificial neural network learns well enough the information associated with the data of training, however, the capacity of it is lost for the estimation of the values of outputs in proportion to input data, which does not consist of the process of training [36]. An early-stop procedure [37] is performed to escape this unwanted problem, which includes the definition of a validation sample and the highest iteration number for the early-stop process; beginning the process of training [38]. Evaluate the model operation on the validation sample when the biases and weights have been updated in each iteration [39]. The training will stop if this operation faces an increase in error over the predetermined epochs’ number in (1). Notably, in the process of training, there is no interference of the validation sample in the adjustment of biases and weights; and the adoption of the iteration parameters that are reporting the optimum operation over the sample of validation.

### 2.6 Algorithm: coot optimizer (CO)

The rails, or Rallidae, are a large family of birds that live on the ground. Coots is a member of this family. On their forehead, there are decorations like prominent frontal shields. Coots have different behaviors on the surface of the water. To present a new optimization process in this paper, this bird's behavior on the surface of the water is considered. Three movements are included in the coot crowd behavior on the water [40]. A disordered movement of an activity, a synchronized movement, and a chain move on the water surface [41]. They have various collective behaviors, which are simulated herein. The whole swarm is headed in the direction of the goal by some coots in front of the swarm that is considered swarm leaders.

Then, four various moves of coots on the water surface are considered here as defined in the following.

- Randomly moving from one side to another side
- Chain movement
- Adjust the location by the swarm leaders
- Lead the swarm by the leaders in the direction of the optimum region

***Mathematical model;*** all algorithms have a similar optimization framework. The algorithm begins with primary random individuals, which are repeatedly assessed by the objective function, and an optimum value is set. Also, based on a rule set this is enhanced [42]. It is not validated to obtain a solution in a single run due to the optimization methods based on population search for the desired value of the optimization problems [43]. Nevertheless, it is more probable to obtain the global optimum with enough amounts of optimization iterations and random solutions. The random population is created by the following equation:

(9) CootPos(i)=rand(1,d).*(ub−lb)+lb

where, CootPos(i) defines the location of the coot. d is the variables or problem dimensions number. ub and lb stand for the higher and minimum boundaries of the solution space. This is presented in Eq. (10). Various upper and lower bounds problems may be defined for each variable.

(10) $$lb=\left[{lb}_{1}.{lb}_{2}.\;\ldots .\;{lb}_{d}\right],ub=\left[{ub}_{1}.{ub}_{2}.\;\ldots .\;{ub}_{d}\right]$$

Each solution fitness should be determined by the objective function after the population is initialized and the location of each agent is defined. The *NL* number of coots as swarm leaders are chosen randomly. Now, the aforementioned movements of coots are performed.

*Random movement from one side to another side;* a random location is assumed based on the following equation to perform this movement and cause the coot to move in the direction of this random location.

(11) Q=rand(1.d).×(ub−lb)+lb

Various parts of the solution space are explored by this movement of the coot. To avoid the local optima, this movement can be helpful. The new location of the coot is defined below:

(12) CootPos(i)=CootPos(i)+A×R2×(Q−CootPos(i))

where R2 defines a random value in the range [0, 1]. A can be defined as follows:

(13) $$A=1-L\times \left(\frac{1}{Iter}\right)$$

where L stands for the present iteration and Iter defines the highest iteration.

*Chain movement;* to perform this move, the mean location of 2 birds can be utilized or the distance vector between them can be calculated and then have a movement to the other coot in half. In this section, the first approach is applied and the new location of the coot is defined below:

(14) CootPos(i)=0.5×(CootPos(i−1)+CootPos(i))

where, CootPos(i−1) is the coot number two.

*Adjust the location by the swarm leaders;* some coots lead the swarm in front of the swarm and the others should adjust their location according to the leaders of the swarm and have a movement near them. Each coot can adjust its location according to the average location of the leaders, and in this way, the location of the coots can be updated. For leader selection and for performing this movement, a procedure is defined as follows:

(15) K=1+(iMODNL)

where i defines the present coot's index number. NL stands for the number of leaders. K denotes the index number of the leader. The updated location of the coot according to the chosen leader is defined as follows:

(16) CootPos(i)=LeaderPos(k)+2×R1×cos(2Rπ)×(LeaderPos(k)−CootPos(i))

where, LeaderPos(k) defines the chosen leader's location. R1 stands for a random amount in the range [0, 1]. R refers to a random amount in the range [-1, 1].

*Lead the swarm by the leaders in the direction of the optimum region;* the swarm should be led in the direction of a target (optimum region), so it is required to update the location of the leaders toward the target. This is defined in Eq. (17), which searches for preferable locations near this present optimum point. Leaders should have moved far from the present optimum location to obtain better locations in some cases. A proper way of approaching the optimum position and escaping from it.

(17) $$LeaderPos\left(i\right)=\left\{ \begin{array}{c} D\times R3\times \cos\left(2R\pi \right)\times R4<0.5\\ \left(g_{Best}-LeaderPos\left(i\right)\right)+g_{Best}\\ D\times R3\times \cos\left(2R\pi \right)\times R4>=0.5\\ \left(g_{Best}-LeaderPos\left(i\right)\right)-g_{Best} \end{array}\right.$$

where, $g_{Best}$ defines the best location ever achieved. R3 and R4 refer to random values between 0 and 1. D is defined as follows:

(18) $$D=2-L\times \left(\frac{1}{Iter}\right)$$

2×R3 gives bigger random movements thus it is avoided from trapping into the local optima, i.e., the exploration is also implemented at the exploitation step. cos(2Rπ) searches near the optimum search agent with various radius to obtain a proper location compared to the best location.

All various movements are considered randomly to maintain the optimizers’ random nature, i.e., the coot can have movement randomly, toward swarm leaders or in a chain when performing the algorithm.

*Modified Coot Optimization Algorithm (MCOA);* this algorithm is a newly introduced bio-inspired method in optimization that can be utilized in various problems [44]. However, sometimes this algorithm requires some modifications to avoid premature convergence and local optima [45]. Two modification methods are presented in this paper to solve these problems [46]. Providing self-adaptive weight is the first modification method [47]. To resolve the convergence rate problems of the CO this method can be more efficient [48]. In evaluating the CO, the promising values should have been obtained for the candidates at the primary phase, but in the later phases, the local search is done. A weight is considered to resolve this for adjusting the degree of approaching that is obtained by the following formula:

(19) $$LeaderPos\left(i\right)=\left\{ \begin{array}{c} D\times w\times \cos\left(2R\pi \right)\times R4<0.5\\ \left(g_{Best}-LeaderPos\left(i\right)\right)+g_{Best}\\ D\times w\times \cos\left(2R\pi \right)\times R4>=0.5\\ \left(g_{Best}-LeaderPos\left(i\right)\right)-g_{Best} \end{array}\right.$$

The weight varies adjustably and increases slowly to decrease the difference between the best from the worst solutions so that a good balance is created between exploitation and exploration.

The next modification method is the chaos theory, which has been used to adjust the quality of the optimum solution. The chaotic mechanism is used to resolve premature convergence due to that the results of the local and global values are sometimes equal. An ergodic result is provided by this mechanism to regulate the quality of the solution. In this regard, the logistic map is used. The individual updating is obtained as given below:

(20) $$CootPos\left(i\right)=x_{m+1}\times \left(ub-lb\right)+lb$$

where,

(21) $$x_{m+1}=4x_{m}\left(1-x_{m}\right)$$

where, $x_{m}$ refers to the $m^{th}$ iteration number. m define the number of iterations. $x_{0}$ stands for a primary random amount in the range [0, 1].

***Algorithm validation;*** For result validation of the presented modified coot optimization algorithm, its achievements have been put in comparison with several latest algorithms which are elephant herding optimizer (EHO) [49], Chimp optimization algorithm (ChOA) [50], Firefly algorithm (FA) [51], CO and MCOA [52] to show better results of the suggested optimizer compared to other optimizers. The applied test functions for confirmation are including Ackley, Rastrigin, Sphere, and Rosenbrock. Table 4 states the details of the evaluated standard benchmark functions.

**Table 4:** The evaluated standard benchmark functions detail.

Function	Constraints
$f_{1}\left(x\right)=10D+\sum_{i=1}^{D}\left(x_{i}^{2}-10\;\cos\left(2\pi x_{i}\right)\right)$	[-608, 608]
$f_{2}\left(x\right)=\sum_{i=1}^{D-1}\left(100\left(x_{i}^{2}-x_{i+1}\right)+{\left(x_{i}-1\right)}^{2}\right)$	[-2.982, 2.982]
$f_{3}\left(x\right)=20+e-20\;\exp\left(-0.2\sqrt{\frac{1}{D}\sum_{i=1}^{D}\left(x_{i}^{2}\right)}\right)-\exp\left(\frac{1}{D}\sum_{i=1}^{D}\left(\cos\left(2\pi x_{i}\right)\right)\right)$	[-11.50, 11.50]
$f_{4}\left(x\right)=\sum_{i=1}^{D}x_{i}^{2}$	[-608, 608]

Table 5 states the variable setting of the studied algorithms for the evaluation.

**Table 5:** The parameter setting of the investigated optimizers.

Algorithm	Parameter	Value
EHO [49]	R	1000
	nClan	4
	α	0.25
	β	0.05
	γ	0.02
**ChOA** [50]	$r_{1}$ and $r_{2}$	Random
	m	Chaotic
FA [51]	α	**0.2**
	β	**0.5**
	γ	**1**
**CO and** MCOA [52]	Number of search agents	30

The algorithm is independently run 30 times to find reliable achievements. The dimension of all benchmark functions is set to 30. In addition, the minimum amount of the test functions is equal to 0. This means that the small values of each algorithm show better results compared to the others. The algorithm's efficiency is evaluated by two measurement indicators which are the mean value (M) and the value of the standard deviation (Std). The comparison achievements of the investigated optimizers used for the test functions are stated in Table 6.

**Table 6:** The comparison achievements of the investigated optimizers used to the test functions.

Test function		MCOA	CO [52]	EHO [49]	ChOA [50]	FA [51]
$F_{1}$	M	0.00	3.34	4.21	7.18	3.80
	Std	0.00	2.44	3.57	6.96	2.21
$F_{2}$	M	2.89	4.47	5.09	4.31	3.82
	Std	1.50	3.46	4.16	3.19	2.12
$F_{3}$	M	0.00	5.2e-20	3.5e-13	6.15e-10	4.86e-5
	Std	0.00	0.00	2.7e-13	5.42e-10	3.67e-5
$F_{4}$	M	0.00	0.00	4.9e-7	7.11e-7	6.52e-8
	Std	0.00	0.00	3.54e-7	6.47e-7	5.73e-8

According to this table, the presented MCOA gives the lowest amount for all test functions, which indicates its higher accuracy to achieve the best value. Moreover, the lowest amount for the Std indicates its better consistency to solve the problems. That is to say, however, the algorithm nature is random, but also finally, for various runs, near-optimal values can be obtained.

*Optimized MCOA-ANN*; for the training of the proposed neural network, a metaheuristic-based backpropagation algorithm has been used. Here, we used a modified design of MCOA to deliver better effectiveness with minimum error in the training of the neural network. Indeed, the main target is the minimization of the sum of square error (SSE) between the experimental data and the data achieved by the designed network, in other words:

(22) $$\min\sum (y_{e}-y_{o})$$

where,

(23) $$y_{o}=\sum wx+b$$

where, $y_{e}$ and $y_{o}$ are the experimental and network outputs, respectively.

The termination condition for this network is to reach 1e-4 tolerance for the aforementioned objective function.

Consequently, according to the explained methodology, 7 various meta-models based on the artificial neural network have been developed along with the MCOA algorithm in the optimization process.

*Optimization parameters design;* the design of the MCOA has been implemented on the MATLAB platform. Then, to achieve the Pareto sets by the meta-model-based also optimization methods based on simulation, the MCOA structure is employed.

## 3 Results

This section discusses comprehensive sets of validation achievements for the meta-models based on ANN and multiple-criteria optimization.

**Fig. 8:**
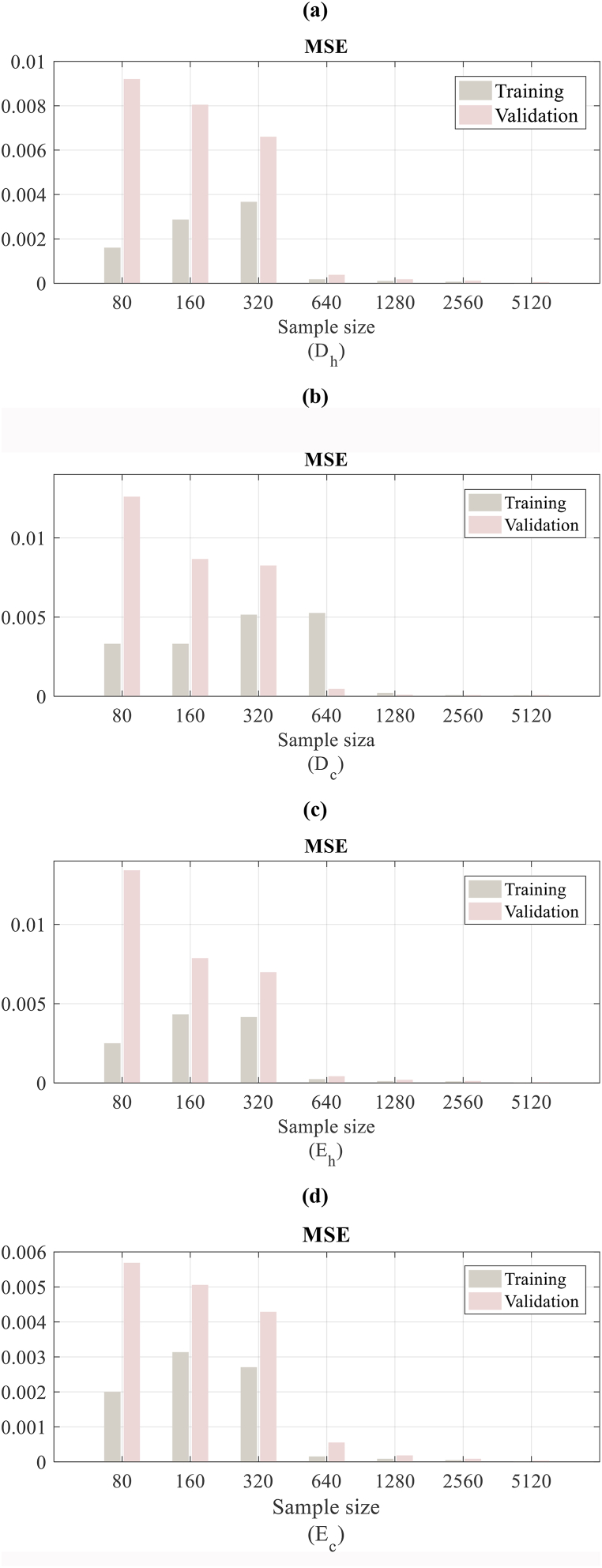
MSE convergence for ANN outputs about the sample size of training. (a: heating degree-hour, b: cooling degree-hour, c: heating energy consumption, d: cooling energy consumption).

*The efficiency of the meta-models based on ANN;* mean squared error convergence for samples of training and validation as well as for the output of artificial neural network considering 7 sample sizes of training is represented in Fig. 8 (a - d). About the mean squared error convergence for training and validation for $D_{h}$ and $D_{c}$, there is the same design: the mean squared error significantly reduces for the sample size of 640 considering former sample sizes of 320, 160, and 80, therefore continuously reducing for the higher sample sizes. The same performance can be observed for $E_{h}$ and $E_{c}$, noting that a sample size of 1280 is required for $E_{c}$ to reduce the mean squared error to the same sequence as the rest outputs.

**Fig. 9:**
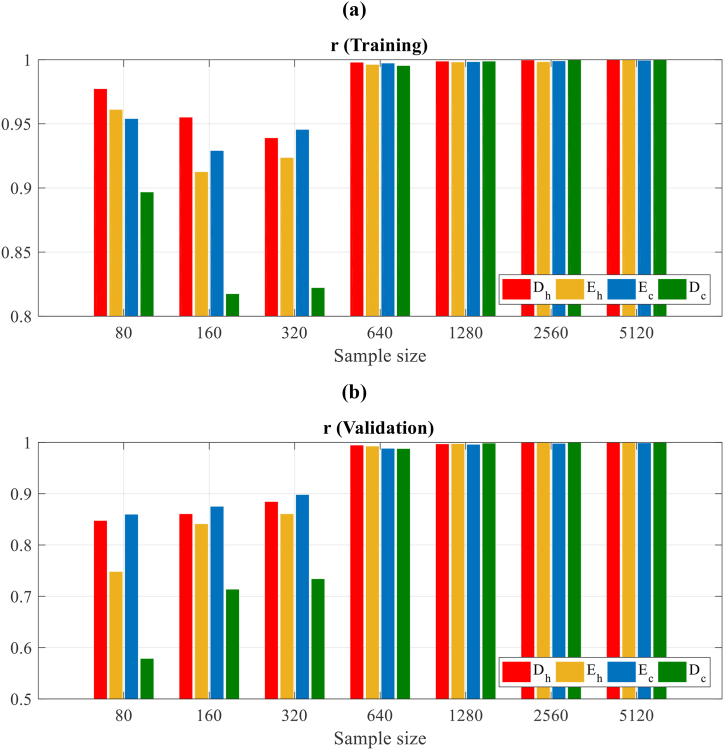
Correlation coefficient convergence for ANN outputs about the sample size of training (a) Training and (b) Validation.

The *r* convergence for each ANN output concerning the size of the sample of training for desegregating the efficiency of the meta-models based on ANN for each output is depicted in Fig. 9. As regards the sample of training, the confirmation is appropriate for all sample sizes, obtaining values higher than 0.95 for $E_{h}$, $E_{c}$, and $D_{h}$, but not for the $D_{c}$, which is lower than 0.9 as shown in Fig. 9 (a,b). This efficiency is slightly lesser for the 160 and 320 sizes of samples, but after it gets higher to 0.98 for all outputs of ANN, it mildly increases continuously for bigger sizes of the sample.

On another side, considering the efficiency of artificial neural network meta-models (ANN-MM) on the samples of validation, it is notable that the estimation for the sample size of 80 is not sufficiently precise for $D_{c}$ with the value of *r* inferior to 0.6 and for $E_{h}$ inferior to 0.75. Therefore, by an increase in the sample size, the efficiency of the artificial neural network is enhanced, however, an acceptable fitting is obtained for sample sizes 640, 1280, 2560, and 5120. This performance shows that while an appropriate behavior on the samples of training with the small size of samples is achieved for an ANN, the behavior on the rest of the representative points is not satisfactory. This is due to an “early stop” procedure to prevent over-fitting on the training sample and using another representative sample made by the LH sampling technique for validation.

*Training sample size effect on the results of Pareto set;* the evaluation of the effect on the results of the Pareto set accuracy achieved with various sizes of sample for the ANN-MM training is presented in this Section.

**Fig. 10:**
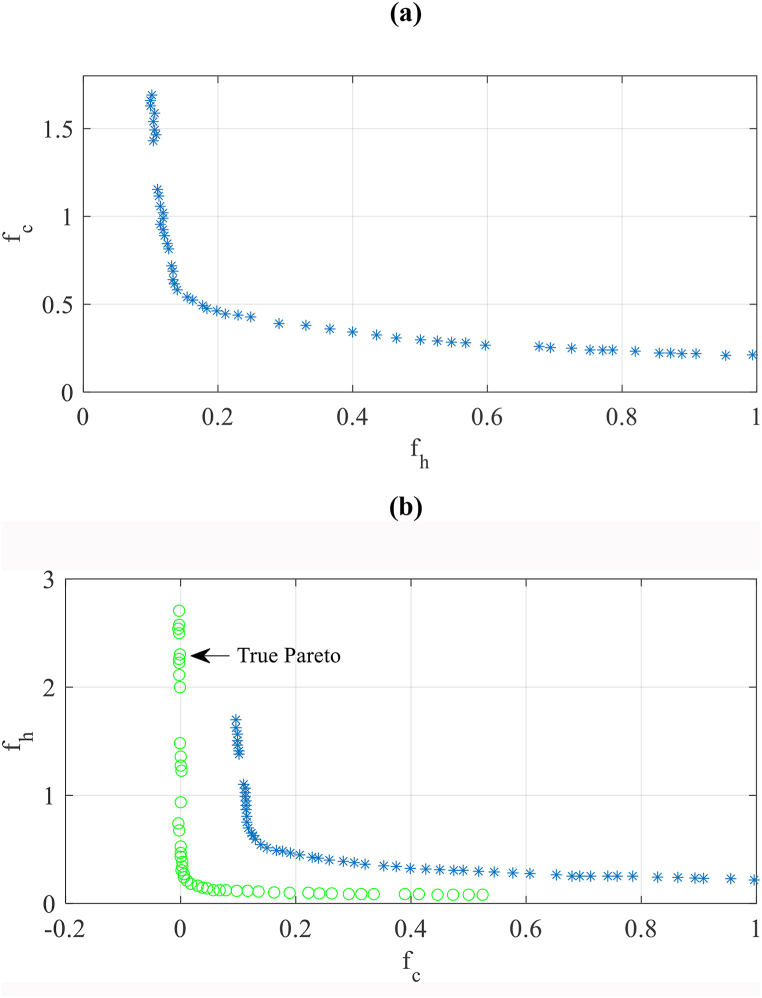
The Pareto set was found by the method based on a meta-model with the ANN-80 compared to the method based on simulation. (a: Pareto set by ANN-80, b: validation of Pareto set by ANN-80).

The Pareto set is obtained when the artificial neural network with the size of 80 meta-models and MCOA is coupled to optimize the case study building as depicted in Fig. 10(a–c). The results seem acceptable regarding the optimum balance (Pareto set) of the case study between cooling and heating efficiency. Nevertheless, as represented in Fig. 10b, when a comparison of these results with the “true” Pareto set obtained by the methodology based on simulation, they have unsatisfactory accuracy compared with the real results. For major evaluation and discussion, the “true” Pareto set achieved by the method based on simulation compared with the Pareto set obtained from the optimization method based on meta-model with other ANN-MM with sample sizes of 160–5120.

**Fig. 11:**
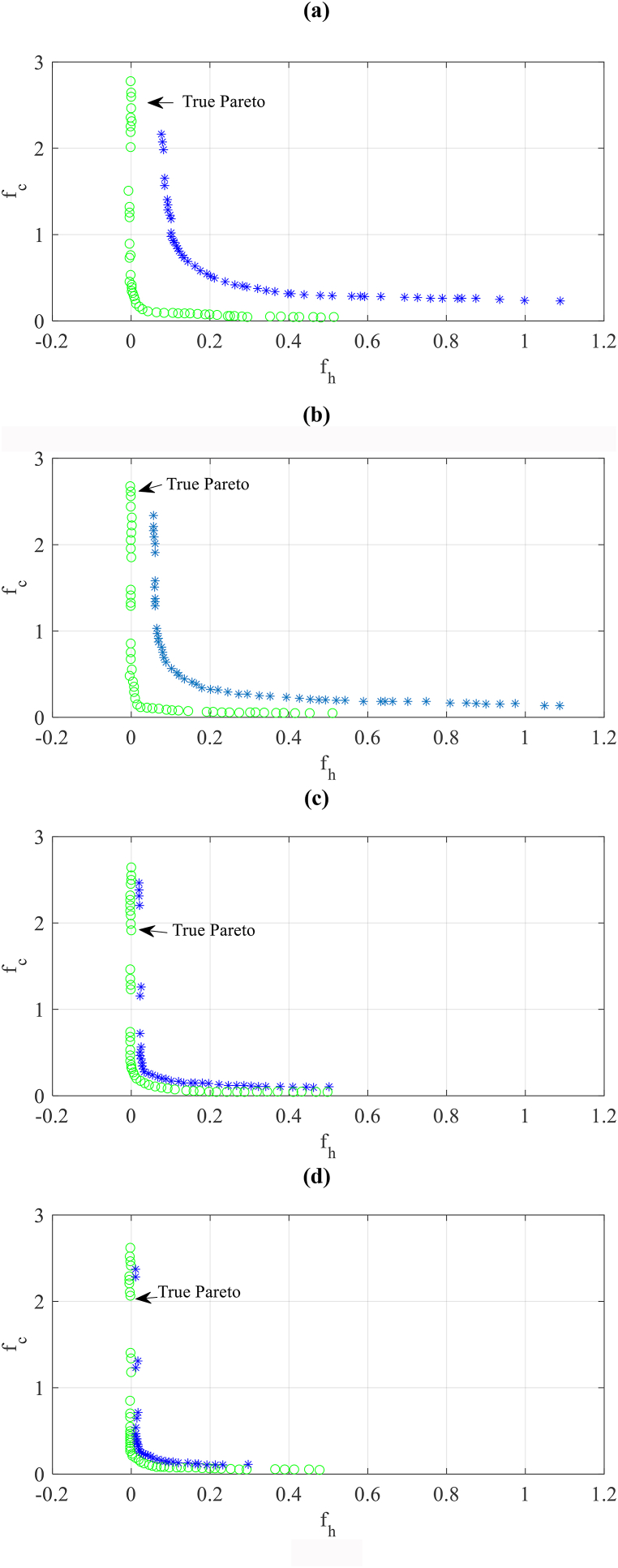

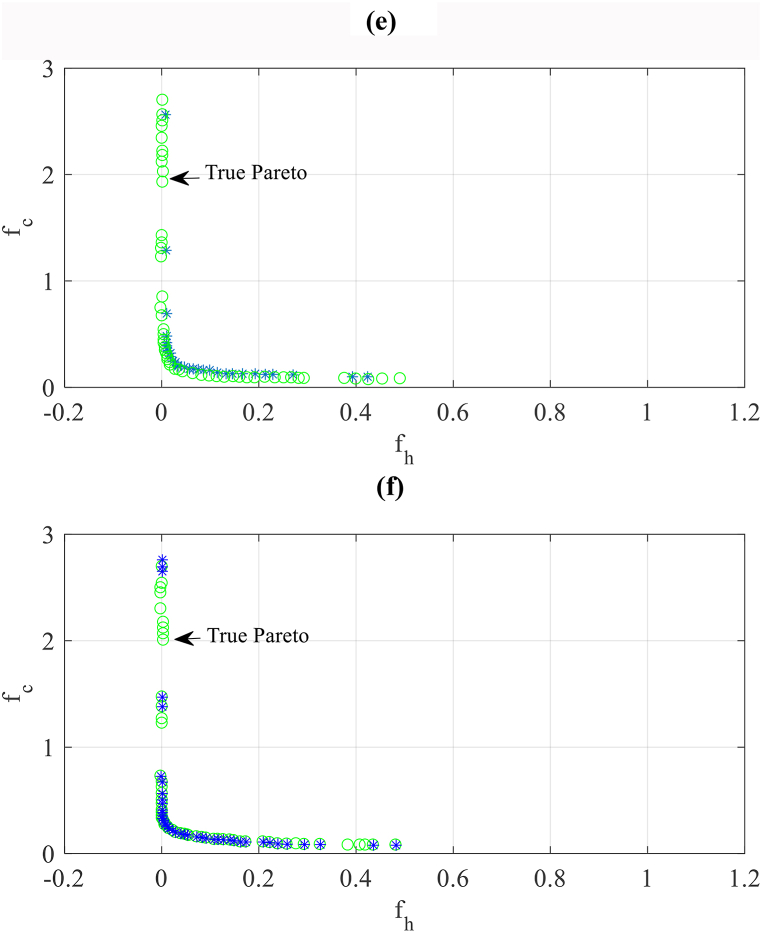
The Pareto set found by the method based on the meta-model with various sample sizes compared with the “true” Pareto set found by the method based on the simulation. (a: ANN-160, b: ANN-320, c: ANN-640, d: ANN-1280, e: ANN-2560, f: ANN-5120).

With 160 and 320 sample sizes of the ANN meta-model, the results depicted in Fig. 11a and b shows that the preciseness to the “true” Pareto set mildly enhances regarding meta-model with an 80 sample size, however, the values and forms of these sets are still slightly distant from the real results. According to Fig. 11c, the accuracy of the results is significantly enhanced and these results are so close to real results for the front obtained by the ANN meta-model trained with 640 samples. An equal result with a rather improvement is achieved for the ANN meta-model with 1280 size. It can be seen that by the achieved results, the present problem can be solved for both cases (640 and 1280).

Finally, as shown in Fig. 11e and f, the achieved results for the ANN meta-models trained with 2560, and 5120 samples are approximately equal to the results obtained by the simulation-based method.

At last, a quantitative evaluation of the effect of the sample size of training on the precision of ANN-MM over the “true” Pareto set has been performed. Then, an inverse validation has been suggested that includes the performance analysis of ANN-MM for each optimum Pareto of the real Pareto set. To perform this, 12 design variables are used that specify each optimum Pareto for feeding up the ANN-MM, to carry out a comparison of the $E_{h}$, $E_{c}$, $D_{c}$ and $D_{h}$ values achieved as outputs of ANN with the ones obtained by the simulations of EnergyPlus™. The root mean square error (RMSE) through the Pareto set is used to measure the accuracy for each output as follows:

(24) $${\text{RMSE}}_{k}=\sqrt{\frac{1}{N}\sum_{i=1}^{N}(O_{i}-D_{i})}$$

where, ${RMSE}_{k}$ is the RMSE for $k=E_{h}$, $E_{c}$, $D_{c}$ or $D_{h}$. $O_{i}$ defines the obtained output value of ANN-MMs for the optimum Pareto i. $D_{i}$ refers to the output value of the experimental data. N denotes the optimum number of Pareto points on the set.

**Fig. 12:**
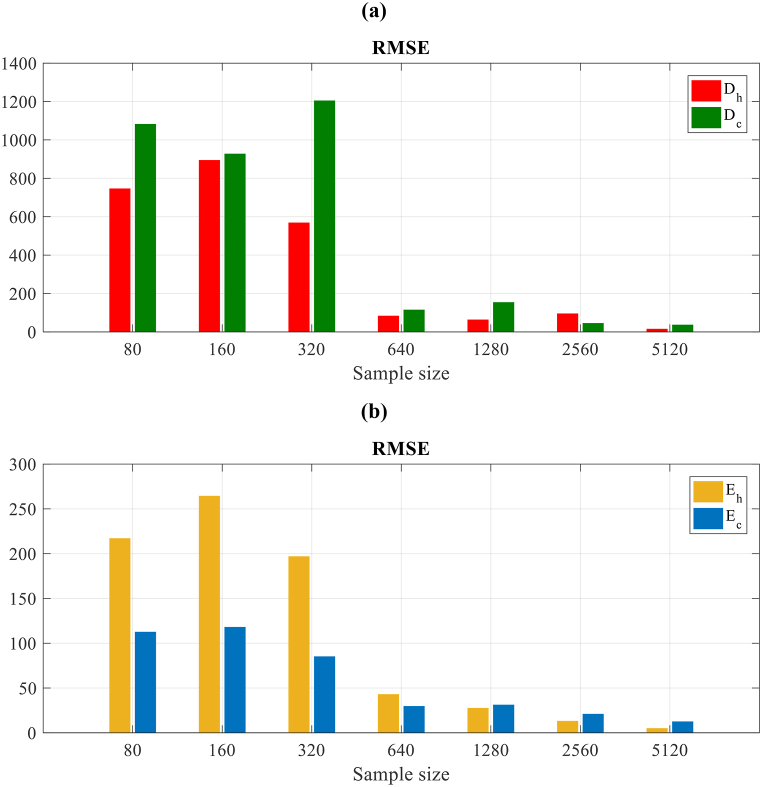
RMSE convergence through the “True Pareto” set for all outputs of ANN concerning the training sample size. (a: degree-hour, b: energy consumption).

The assessment of the RMSE for the sample size of training for the 4 outputs set in the energy use and the degree-hours as represented in Fig. 12. As depicted in Fig. 12a, considering the errors related to degree hours, ${RMSE}_{D_{c}}$ and ${RMSE}_{D_{h}}$, an equal performance is seen, to be high for the minimum sample size of training. Therefore, the errors reduce as the sample size of training increases, obtaining desired low values for the ANN-MM with 640 sample size. For higher sizes, this error reduces continuously. Generally, the equal performance has been seen for this error of energy use, depicted in Fig. 12b, however, the precision for $E_{c}$ is significantly higher compared for $E_{h}$ in lower samples that show the simplicity of predicting this output. Nevertheless, to obtain desired precision on the Pareto set, it is required to have 640 points of training.

## 4 Discussion

Firstly, it is found that to obtain precise results for the Pareto sets of real case study buildings, the small sizes of samples are not adequate. This is seen in Fig. 10, where the Pareto set obtained by the 80 sample size of ANN is not near to the “true” Pareto achieved through the method based on simulation. In the samples of training for real case buildings, 640–1280 points are required to obtain precise results on the Pareto set as shown in Fig. 11, Fig. 12.

It is assumed that 1280 points are adequate in this case study to obtain precise results on the Pareto set. Thus, 75% of the required simulations’ number based on physics has been saved by this size of sample considering the 5120 applied in the method based on simulation. Notably, these sample sizes of training ensure precise Pareto sets agree with the sizes whereat the ANN-MMs gave proper behavior on both samples of training and validation as shown in Fig. 8, Fig. 9.

To validate the achieved results, a comparison of the proposed method with some other methods employed in other studies has been implemented as presented in Table 7.

**Table 7:** The results of the comparison between the applied method and some other techniques.

Algorithm	MCOA	GA [53]	NSGA-II [54]	NSGA-II-ANN [22]
Reduction of the required simulations	75%	68%	57%	44%

As can be observed from Table 7, the proposed method gives better results for the reduction of the required building performance simulations in comparison to the other introduced methods, which validates the accuracy and reliability of the presented method.

According to the present results, concerning the stopping criteria of the suggested meta-model generation method, we can deduce that appropriate stopping criteria should estimate the efficiency of the artificial neural network through the sample of validation and for all outputs of the ANN, make the correlation coefficient *r* proper then. This has been approved by Fig. 9, where the ANN-MMs give an appropriate efficiency for small sizes of the sample with r>0.8, but not for the samples of validation. Notably, it is required for the sample of validation to be the design space representative, as it has been assumed in this study, creating another sample, which is independent, by the Latin hypercube sampling approach.

For developing ANN-MM with a few simulations based on physics and for obtaining precise results in multiple-criteria optimization problems of the building this method is suggested that solves the shortcomings of classic Trial and Error methods. Nevertheless, it is globally considered that the efficiency of the meta-model is enhanced in the overall design space following each increase in the sample size of the training. While this is accepted in the present utilization, for other applications such as energy labeling of the building [55] it is flawless. It is needed for an optimum meta-model to have average global precision but a proper efficiency on the Pareto set for multiple-criteria optimization. Therefore, the number of needed simulations based on physics can be decreased by iterative methods with a smart sampling technique.

## 5 Conclusions

An effective approach has been proposed in this paper to find a solution for multi-criteria building performance optimization problems by a new method by a meta-model for the minimization of the required overall number of building performance simulations based on physics. EnergyPlus™ tool was used for the building performance simulation, then a couple of the multi-criteria Modified Coot Optimization Algorithm (MCOA) dynamically combined with the artificial neural network meta-models (ANN-MM) was employed. As a novelty, a dedicated method was used for the sample generation for ANN-MM training and validation for building energy simulations’ number minimization and validating the accuracy of the optimization results. This approach has been applied for the thermal comfort and energy efficiency optimization of a real house to achieve the optimum balance between the heating/cooling behaviors of the case study building. Moreover, the results were compared with the “true” Pareto set achieved by an optimization technique based on simulation for more validation. According to the results, the proximity of the building designs on the Pareto set achieved via a method based on a meta-model concerning the “true” Pareto sets was overall correlated with the training sample size. It should be noted that the size of samples is related to the type of utilization. The proposed method could decrease up to 75% of the required building performance simulation to achieve the Pareto set of a real multi-criteria building performance optimization problem with accurate results. To develop ANN-MM with several physics-based simulations and for obtaining precise achievements in multiple-criteria optimization problems of the building this approach was presented that solves the shortcomings of classic Trial and Error methods. It is needed for an optimum meta-model to have average global precision but a proper efficiency on the Pareto set for multiple-criteria optimization. Therefore, the number of needed simulations based on physics can be decreased by iterative methods with a smart sampling technique. Furthermore, the design of the method based on the meta-model can be considered in future works where the method and training sample of it, are developed iteratively. A smart sampling method can be used to obtain the Pareto set, fast and highly decrease the number of needed building performance simulations based on physics. These also can be recommendations for future researchers.

## Author contribution statement

Xiaoming You: analyzed and interpreted the data; performed the experiments; Gongxing Yan: contributed reagents, materials, analysis tools or data; conceived and designed the experiments; Myo Thwin: wrote the paper; conceived and designed the experiments.

## Data availability statement

The authors do not have permission to share data.

## Declaration of competing interest

The authors declare that they have no known competing financial interests or personal relationships that could have appeared to influence the work reported in this paper.

## Nomenclature

### Symbols

$f_{a}$: Particular objective
y: Defines *k* building design variables set
$T_{l}$: The lower acceptable temperature
$T_{o}$: The operative temperature in the room at time t
$T_{u}$: The upper acceptable temperature
$T_{pma\;\left(o\right)}$: The prevailing mean outdoor temperature
$E_{c}$: The yearly energy consumption of the air conditioners for cooling
$E_{h}$: The yearly energy consumption of the air conditioners for heating
$w_{d}$ & $w_{e}$: The weighting factors where $w_{d}+w_{e}=1$
$z_{0}$: The design variables set for $C_{0}$. $w_{d}=w_{e}=0.5$
$T_{ex}$: The exterior temperature
$T_{in}$: The interior temperature
$D_{h}$: The discomfort index by the heating degree-hour
$D_{c}$: The discomfort index by the cooling degree-hour
$f_{c}$ & $f_{h}$: The weighted sum of sub-objectives $D_{c}$, $D_{h}$, $E_{c}$ and $E_{h}$
$V_{1}-V_{7}$: The selected continuous variables
$V_{8}$-$V_{12}$: The categorical design variables
CootPos(i): The location of the coot
d: The variables or problem dimensions number
ub & lb: The higher and minimum boundaries of the solution space
R2: A random value in the range [0, 1]
L: The present iteration
i: The present coot's index number
NL: The number of leaders
K: The index number of the leader
LeaderPos(k): The chosen leader's location
R1: A random amount in the range [0, 1]
R: A random amount in the range [-1, 1]
$g_{Best}$: The best location ever achieved
R3 & R4: Random values between 0 and 1
$x_{m}$: The $m^{th}$ iteration number
m: The number of iteration
$x_{0}$: A primary random amount in the range [0, 1]
$y_{e}$ & $y_{o}$: The experimental and network outputs
${RMSE}_{k}$: The RMSE for $k=E_{h}$, $E_{c}$, $D_{c}$ or $D_{h}$
$O_{i}$: The obtained output value of ANN-MMs for the optimum Pareto i
$D_{i}$: The output value of the experimental data
N: The optimum Pareto points number on the set

### Abbreviations

GHG: Green House Gas
BPS: Building Performance Simulation
MM: Meta-Model
SM: Surrogate Model
PCMs: Phase-Change Materials
GMDH: Grouped Method of Data Handling
BPO: Building Performance Optimization
ANN: Artificial Neural Network
ALH: Augmented Latin Hypercube
MCOP: Multiple-Criteria Optimization Problem
MCOA: Modified Coot Optimization Algorithm
PTHPs: Packaged Terminal Heat Pumps
T&V: Training and Validation
LH: Latin Hypercube
EHO: Elephant Herding Optimizer
ChOA: Chimp Optimization Algorithm
FA: Firefly Algorithm
CO: Coot Optimizer
M: Mean Value
Std: Standard Deviation
SSE: The sum of Square Error
ANN-MM: Artificial Neural Network Meta-Models
RMSE: Root Mean Square Error
MSE: Mean Square Error
